# Effects of Bovine Colostrum with or without Egg on In Vitro Bacterial-Induced Intestinal Damage with Relevance for SIBO and Infectious Diarrhea

**DOI:** 10.3390/nu13031024

**Published:** 2021-03-22

**Authors:** Raymond J. Playford, Naheed Choudhry, Paul Kelly, Tania Marchbank

**Affiliations:** 1 Centre of Immunobiology, Blizard Institute, Barts and The London School of Medicine, Queen Mary, University of London, London E1 2AT, UK; n.choudhry@qmul.ac.uk (N.C.); m.p.kelly@qmul.ac.uk (P.K.); t.marchbank@qmul.ac.uk (T.M.); 2 Research & Development, Pantheryx Inc., Boulder, CO 80301, USA

**Keywords:** small intestinal bacterial overgrowth (SIBO), nutraceuticals, repair, antimicrobial, irritable bowel syndrome, leaky gut

## Abstract

Small intestinal bacterial overgrowth (SIBO) occurs commonly, is difficult to treat, and frequently recurs. Bovine colostrum (BC) and chicken eggs contain immunoglobulins and other components that possess antimicrobial, immunoregulatory, and growth factor activities; however, it is not known if they have the ability to reduce injury caused by the presence of bacteria associated with SIBO (*Streptococcus*, *Escherichia coli*, *Staphylococcus*, *Bacteroides*, *Klebsiella*, *Enterococcus*, and *Proteus*) and infectious diarrhea (enteropathogenic *Escherichia coli*, *Salmonella*). We examined the effects of BC, egg, or the combination, on bacterial growth and bacteria-induced changes in transepithelial electrical resistance (TEER) and bacterial translocation across confluent Caco-2 monolayers. BC, egg, or the combination did not affect bacterial growth. Adding bacteria to monolayers reduced TEER and (with minor variations among species) increased bacterial translocation, increased monolayer apoptosis (increased caspase-3 and Baxα, reduced Bcl2), increased intercellular adhesion molecule 1 (ICAM-1), and reduced cell adhesion molecules zonulin1 (ZO1) and claudin-1. BC, egg, or the combination reduced these effects (all *p* < 0.01) and caused additional increases in vascular endothelial growth factor (VEGF) and Heat Shock Protein 70 (Hsp70) expression. We conclude that BC ± egg strengthens mucosal integrity against a battery of bacteria relevant for SIBO and for infectious diarrhea. Oral BC ± egg may have clinical value for these conditions, especially SIBO where eradication of precipitating organisms may be difficult to achieve.

## 1. Introduction

Small intestinal bacterial overgrowth (SIBO) is defined as the presence of excess bacteria within the small intestine. Symptoms associated with SIBO include bloating, flatulence, diarrhea and abdominal discomfort [1]. Many of the clinical features are similar to those of patients with irritable bowel syndrome (IBS), although both conditions may coexist in the same patient [2]. For example, in a series of 87 adult patients presenting with chronic diarrhea, SIBO was subsequently diagnosed as the cause in half the patients, whereas IBS accounted for only 13% [3]. Similarly, Pimental et al. reported that up to 78% of patients with a clinical diagnosis of IBS had a positive lactulose hydrogen breath test, compatible with a diagnosis of SIBO. The extent to which these patients had a “misdiagnosis” of IBS or that the two conditions coexisted in the same patients is unclear, although successful eradication of microbes with antibiotic treatment led to reduced symptoms in about half the patients [4]. Severe SIBO can manifest as malabsorption syndromes, resulting in weight loss, specific nutritional deficiencies, and more generalized complications such as osteoporosis [1]. In less developed countries, up to two-thirds of the children living in urban slums have documented SIBO and this has contributed to the development of environmental enteropathy and stunted growth [5].

Risk factors for development of SIBO include altered intestinal anatomy, for example, blind loop syndrome or presence of a stricture, and medical conditions such as portal hypertension, pancreatic insufficiency, chronic renal failure, hypothyroidism, Crohns’ disease with small-bowel strictures, and any condition causing impaired gut motility. In support of this idea, SIBO is a frequent cause of diarrhea and malabsorption in elderly patients who have developed age-related small bowel dysmotility. Medications known to increase the risk of SIBO includes opiates (through affecting gut motility) and gastric acid suppressants such as proton pump inhibitors [1].

A mixed population of bacteria is often found in patients with SIBO, with some of the commonest species being aerobes such as *Streptococcus*, *Escherichia coli*, *Staphylococcus,* and *Klebsiella* and anaerobes such as *Bacteroides*, *Lactobacillus,* and *Clostridium* [1,6]. The presence of SIBO is often associated with inflammatory changes in the small bowel mucosa including blunting of the villi, atrophy of mucosa and crypts, elevation in the number of intraepithelial lymphocytes, and increased gut permeability [7], with much of the mucosal changes, at least partially, reversing if the microbes are eliminated. Currently recommended medical treatment of SIBO is with nonabsorbable antibiotics, such as rifaximin, with or without probiotics. Unfortunately, treatment with antibiotics is only effective in about 70% of patients [8] and there is a high risk of SIBO relapse in approximately 50% of cases 12 months after initial treatment [9], which requires repeated courses or continuous cyclical use of multiple antibiotics [1]. Although probiotics are sometimes advocated, they have not been conclusively shown to be beneficial and have not reached the evidential threshold to merit recommendation in the latest guidelines of the American College of Gastroenterology [10]. Therefore, novel therapies to address microbial infections and/or mitigate their effects on gut mucosa would be of value.

Currently, there is public demand for more natural types of products, in particular, when required for prolonged usage. Natural products with pharmaceutical activity are sometimes termed nutraceuticals (from nutrition and pharmaceuticals). Two nutraceutical products which have potential value for the treatment of SIBO are bovine colostrum (BC) and chicken egg, used individually or together. BC is the milk produced during the first few days after birth and is a rich natural source of macro- and micronutrients, growth factors, immunoglobulins (particularly IgG), and peptides with antimicrobial activity (e.g., lactoperoxidase). It is produced by the milk industry and sold commercially to promote both human and veterinary general health and immune support. There is also increasing evidence that BC may be of value for the treatment of a variety of medical conditions in children and adults such as gut injury caused by nonsteroidal anti-inflammatory drugs or chemotherapy, necrotizing enterocolitis, and inflammatory bowel disease [11,12], and as a supplement for athletes to aid exercise performance and recovery [13].

Chicken eggs form an important dietary source of calories, protein, fats, and minerals. In addition to their nutritional value, eggs contain many proteins and peptides of therapeutic interest. These include antimicrobial and immunomodulatory factors such as IgY, lysozyme, avidin, ovalbumin, and ovomucoid, which suggests that egg may be a useful natural source of bioactives for clinical use [14,15,16]. Egg has been shown to stabilize gut mucosa against noxious agents, for example, oral egg powder has been shown to reduce DSS-induced colonic injury in mice and NSAID-induced gastric damage in rats [17]. Synergistic responses were seen when the egg and BC were used in combination in the DSS and NSAID models and in in vitro models of proliferation and migration [17]. Further evidence for the value of a BC and egg combination comes from a study performed in Guatemala that showed this combination reduced duration of diarrhea in patients with infectious diarrhea, although interpretation is limited because the study did not examine the individual components (BC and egg) in isolation [18].

To begin to examine the potential value of BC and/or egg to maintain mucosal integrity in patients with SIBO, we performed a series of studies that examined (1) whether these products had bacteriostatic/bactericidal activity against the microbes commonly seen in SIBO and against two common causes of severe infectious diarrhea, namely enteropathogenic *Escherichia coli* (EPEC) and *Salmonella*; (2) whether mucosal integrity of human intestinal cell monolayers was disrupted by the presence of these bacteria administered to the apical surfaces; (3) whether the co-presence of egg, BC, or the combination influenced the damaging effects of the bacteria on the monolayers; and (4) the molecular pathways through which any protective effects may have been mediated.

## 2. Materials and Methods

### 2.1. Bovine Colostrum (BC) and Egg Samples

The pasteurized BC powder (45/15 ColostrumOne^TM^) and a commercial chicken whole egg powder were provided by Pantheryx Inc. (Boulder, CO, USA). BC was collected during the first 24 h post calving and the subsequent powder produced was comprised of 48 g protein, 16 g fat, and 25 g carbohydrate per 100 g of powder and 15 g IgG/100 g powder. The approximate content of major growth factors (based on [19]), were as follows: insulin-like growth factor-1 (IGF-1, 133 ng/mg powder), epidermal growth factor (EGF, 14 ng/mg powder), and transforming growth factor β (TGFβ, 8 pg/mg powder).

The egg powder was comprised of 51 g protein, 43 g fat, and 1 g carbohydrate per 100 g and approximately 1 g IgY per 100 g egg. Ovomucoid content was approximately 50 mg/mg and ovalbumin 400 μg/mg powder, based on [20]. The BC and egg combination were used at a ratio of 60:40, based on the beneficial synergistic effects against DSS injury demonstrated by us previously [17].

### 2.2. Cell Line

Caco-2 is derived from the colorectal adenocarcinoma of a 72-year-old male (ATCC HTB37^TM^, ATCC, LGC standards, Teddington, UK). These were chosen because they are of human gastrointestinal origin and exhibit tight junctions and desmosomes between adjacent cells, therefore, they grow as polarized monolayers which can be used to perform transepithelial electrical resistance (TEER) analyses [21].

### 2.3. Bacterial Strains and Culture

*Escherichia coli* (*E. coli,* ATCC 25922 O6) and *Enterococcus faecalis* (ATCC 29212) were obtained from LGC Standards (Teddington, Middlesex, UK). *Staphylococcus aureus* (NCTC12981), *Streptococcus pneumonia* (NCTC 12695), *Klebsiella pneumoniae* (NCTC 9633), and *Proteus mirabilis* (NCTC 13376) *Salmonella enterica* subsp. *enterica serovar Typhimurium* (ATCC^®®^ 14028™) were obtained from Culture Collections (Public Health England, Porton Down, Salisbury, UK). Enteropathogenic *E. coli* (EPEC, ICC481—O127:H6) was a gift from Gad Frankel at Imperial College London, UK. Nonpathogenic *Escherichia coli* K12 were a gift from Dr David Wareham (Centre for Immunobiology, Blizard Institute, QMUL, London, UK).

Bacterial colonies were stored on Luria-Bertani (LB) agar or on Columbia agar with horse blood (blood agar, Fisher Scientific, Loughborough, UK), and fresh colonies were re-cultured weekly. Before each experiment, a single colony was cultured in LB broth and grown shaking overnight at 37 °C followed by OD600 nm measurement.

### 2.4. Study Series 1: Effect of BC, Egg, or the Combination on Bacterial Growth

BC alone or egg alone (tested at 0.5, 1, 5, and 10 mg/mL) or BC with egg combination (tested at a 60:40 ratio with a final combined concentration of 0.5, 1, 5, and 10 mg/mL) were added to 1 × 10^6^ colony-forming units (CFU)/mL of each pathogen in LB broth for 24 h. Following incubation, samples were serially diluted in PBS and cultured on blood agar and MacConkey agar plates (Fisher Scientific, Loughborough, UK), at 37 °C overnight. Then, the number of colonies formed were determined.

### 2.5. Study Series 2: Effect of BC, Egg, or the Combination on Transepithelial Passage of Bacteria and Transepithelial Electrical Resistance (TEER)

We examined the effect of BC alone, egg alone, or the combination treatment on pathogen-induced transepithelial permeability of confluent polarized Caco-2 monolayers, using two different, previously published, methods. One determined the change in transepithelial electrical resistance, a measure of the barrier property of the epithelium to passive ion movement, where decreased resistance indicates an increase in permeability [22]. The other analyzed bacterial translocation (determined by the number of colony-forming units obtained from medium collected from the basal side of the monolayers) [23].

Monolayers of Caco-2 polarizing colonic adenocarcinoma cells were grown to confluence in DMEM containing 10% FCS, 4 mM L-glutamine, 1000 U/mL penicillin, 100 μg/mL streptomycin, and 1% non-essential amino acids (Invitrogen Life Technologies, Paisley, UK). Cells were grown in 24-well plates holding polyethylene terephthalate (0.4 μm) cell culture inserts (transwell inserts, Millipore, Hertfordshire, UK) and grown until polarization (15–21 days). Formation and disruption of polarized monolayers (membrane integrity) were determined by daily measurement of transepithelial electrical resistance using electrodes (TER) (Millicell-ERS, Millipore, Livingston, UK). The value obtained from a blank insert (with culture medium only) was subtracted to give the net sample resistance, which was, then, multiplied by the membrane area to give the resistance in area-corrected units (Ω/cm^2^). When a consistent mean resistance of >300 Ohms/cm^2^ was obtained (approximately 15–20 days), monolayers were washed three times in medium without antibiotics. Then, BC alone (1 mg/mL), egg alone (1 mg/mL), or BC + egg combination treatments (0.6 mg/mL BC + 0.4 mg/mL egg) and bacteria (1 × 10^6^ CFU/well) were added to the apical surface in culture media without antibiotics or FCS. TEER was measured just prior to addition of bacteria + test products and 24 h later. Bacterial translocation was determined by culturing medium from the basolateral compartment of a transwell insert, after infection for colony quantification for 24 h. Measurements were taken from 6 wells per treatment, results are expressed as mean ± SEM.

### 2.6. Study Series 3: Mechanisms of Action of BC, Egg, or the Combination for Maintaining Epithelial Integrity

Having shown that BC, egg, or combination products reduced monolayer permeability, cleared lysates from in vitro monolayer studies were analyzed to examine possible modes of action.

#### 2.6.1. Cell Lysate Preparation

Following incubation with bacteria ± BC, egg, or the combination, cells were washed in ice-cold PBS, lysed in lysis buffer (50 mM HEPES, 5 mM DTT, 0.1 mM EDTA, 0.1% CHAPS, pH 7.4) for 5 min on ice. Lysates were cleared by centrifugation at 10,000× *g* for 10 min at 4 °C. Protein concentrations were determined by a standard BCA method (Pierce).

#### 2.6.2. Tight Junction Proteins

ZO1 and claudin-1 concentrations were determined using previously published methods [24] and standard ELISA kits (Generon, Slough, UK)

#### 2.6.3. Cell Apoptosis Assays

Active caspase-3 was determined, using methods described previously [24], using commercial colorimetric assay kits (BF3100, R&D Systems, Minneapolis, MN, USA). Concentrations of the anti-apoptotic protein Bcl2 and the proapoptotic protein Baxα were determined in the same cell lysates as used for caspase analyses, using Duoset Elisa kits (R&D Systems Europe Ltd., Abingdon, UK).

#### 2.6.4. ICAM-1, VEGF, and Hsp70

ICAM-1, VEGF, and Hsp70 concentrations in the cleared cell lysates was determined using Duoset Elisa kits, as per the manufacturer’s instructions (R&D Systems Europe Ltd., Abingdon, UK).

### 2.7. Statistical Analyses

All results are expressed as mean ± SEM. Statistics were performed using Graphpad Prism 8 version 8.3.1. The test for normality of data, the Shapiro–Wilks test, was performed and showed equal variances among products. Results were analyzed using a one-way repeated measures analysis of variance (ANOVA). Comparisons between treatments was performed using a Tukey’s multiple comparison test.

## 3. Results

### 3.1. Study Series 1: Effect of BC, Egg or the Combination on Bacterial Growth

Treatment with BC, egg, or the combination did not influence proliferation of any of the bacteria at any of the concentrations tested (Table S1).

### 3.2. Study Series 2: Effect of BC, Egg, or the Combination on Transepithelial Passage of Bacteria and TEER

#### 3.2.1. Bacterial Translocation

**Figure 1:**
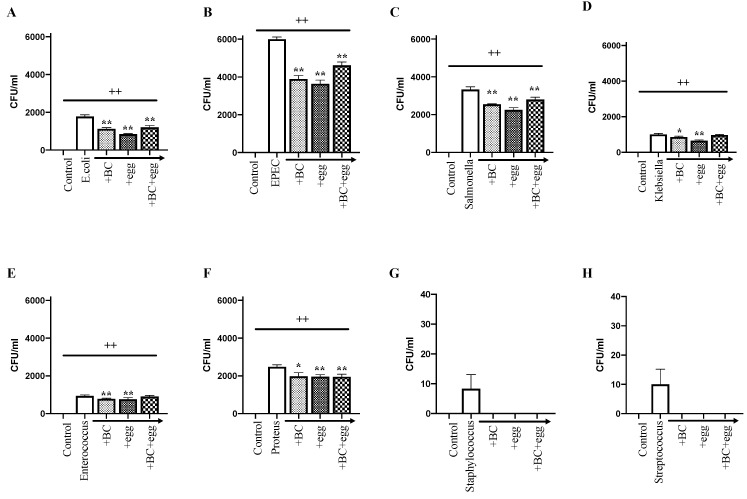
Effect of bovine colostrum (BC), egg, or the combination on transepithelial passage of bacteria. Bacterial strains (1 × 10^6^ colony-forming units (CFU)/well) ± BC (1 mg/mL), egg (1 mg/mL), or the combination (0.6 mg/mL BC + 0.4 mg/mL egg) were added to the apical side of confluent monolayers of Caco-2 cells grown in transwell plates. The medium was collected from the basal side, 24 h later, and assessed for the number of colony-forming units (CFU)/mL. Control wells received no bacteria or test product. (**A**) *Escherichia coli* ATCC 25922 O6; (**B**) enteropathogenic *E. coli* (EPEC); (**C**) *Salmonella*; (**D**) *Klebsiella*; (**E**) *Enterococcus*; (**F**) *Proteus*; (**G**) *Staphylococcus*; (**H**) *Streptococcus.* Note that the scale of the *y*-axis of results for *Staphylococcus* and *Streptococcus* are lower than for other strains. Results are expressed as mean ± SEM for 6 wells. ++ signifies *p* < 0.01 vs. non treated (without bacteria or test product) control, * and ** signify *p* < 0.05 and 0.01 vs. bacteria alone. The nonpathogenic and noninvasive *E. coli* K12 did not result in any CFUs in this experiment, and therefore is not shown.

Figure 1 demonstrates the effects of BC, egg, or the combination on the amount of bacterial translocation across the monolayers for the eight bacteria associated with SIBO or infectious diarrhea.

The nonpathogenic *Escherichia coli* K12 (noninvasive negative control) did not translocate across the monolayer (or affect TEER or any of the other measured parameters). All other strains caused bacterial translocation through the monolayer into the basal medium, with the co-presence of BC, egg, or the combination significantly reducing the number of bacteria subsequently isolated from the basal medium (Figure 1). The highest levels of translocation were seen using EPEC. Lower levels of translocation were seen when testing *Staphylococcus* and *Streptococcus*, with the co-presence of test products completely preventing their translocation (Figure 1).

#### 3.2.2. TEER

**Figure 2:**
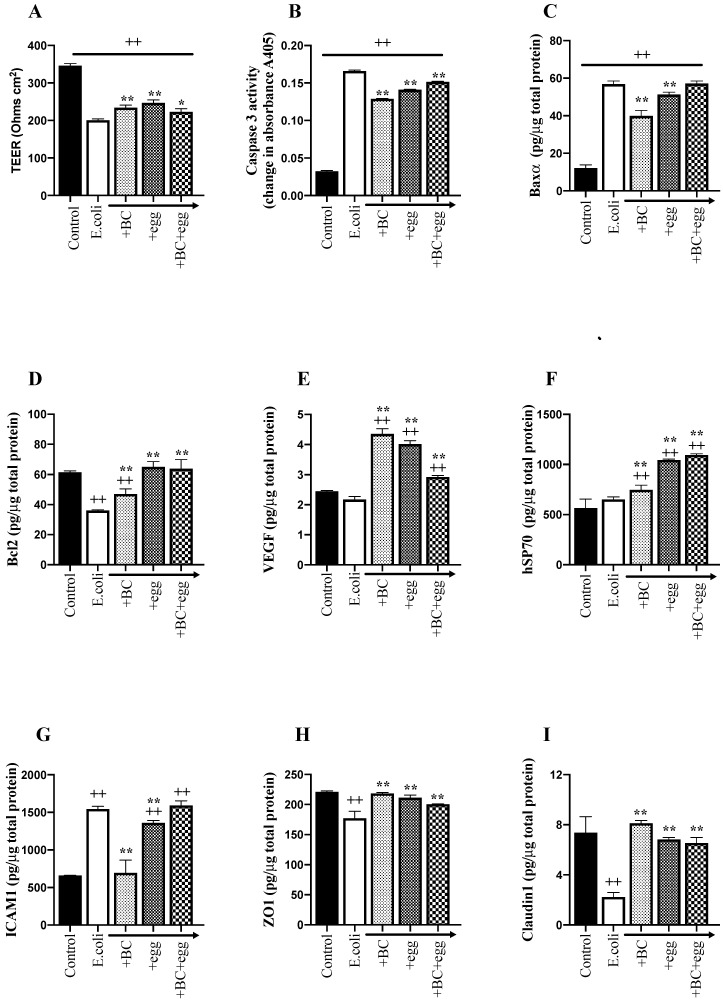
Effect of *Escherichia coli* ATCC 25922 O6 on Caco-2 monolayer TEER and damaging and repair pathways in the presence of BC, egg, or the combination. *E. coli* (1 × 10^6^ CFU/well) ± BC, egg, or the combination was added to confluent Caco-2 cells; 1 mg/mL of BC alone, 1 mg/mL egg alone or 0.6 mg/mL BC + 0.4 mg/mL egg were used. Changes in TEER (**A**) were determined 24 h later. Following incubation, cleared cell lysates were collected and changes in caspase-3 (**B**), Baxα (**C**), Bcl2 (**D**), VEGF (**E**), Hsp70 (**F**), ICAM-1 (**G**), ZO1 (**H**), and claudin-1 (**I**) were determined. Results expressed as mean ± SEM for 6 wells. ++ signifies *p* < 0.01 vs. non treated (without bacteria or test product) control, * and ** signify *p* < 0.05 and 0.01 vs. presence of bacteria alone. The other bacterial strains tested gave similar results (Table 1, Table 2 and Table 3).and are noted by exception in the text.

**Figure 3:**
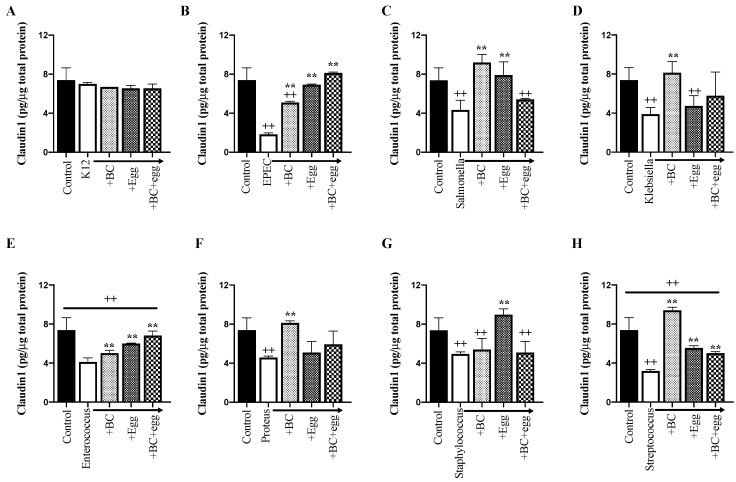
Effect of BC, egg, or the combination on tight junction protein, claudin-1. Bacterial strains (1 × 10^6^ CFU/well) ± BC (1 mg/mL), egg (1 mg/mL), or the combination (0.6 mg/mL BC + 0.4 mg/mL egg) were added to confluent monolayers of Caco-2 cells. Cleared lysates were prepared 24 h later. Control wells received no bacteria or test product. (**A**) *Escherichia coli* K12 (negative control); (**B**) EPEC; (**C**) *Salmonella*; (**D**) *Klebsiella*; (**E**) *Enterococcus*; (**F**) *Proteus*; (**G**) *Staphylococcus*; (**H**) *Streptococcus.* Results are expressed as mean ± SEM from 3 wells. ++ signifies *p* < 0.01 vs. non treated (without bacteria or test product) control and ** signifies *p* < 0.01 vs. bacteria alone.

**Figure 4:**
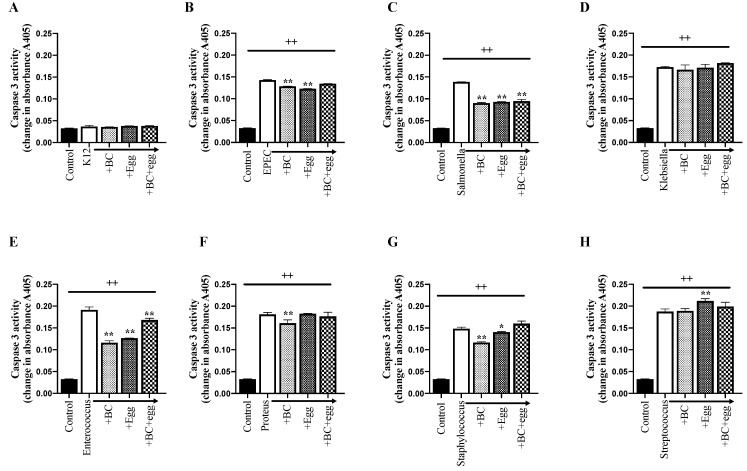
Effect of BC, egg, or the combination on active caspase-3. Bacterial strains (1 × 10^6^ CFU/well) ± BC (1 mg/mL), egg (1 mg/mL), or the combination (0.6 mg/mL BC + 0.4 mg/mL egg) were added to confluent monolayers of Caco-2 cells. Cleared lysates were prepared 24 h later. Control wells received no bacteria or test product. (**A**) *E. coli* K12 (negative control); (**B**) EPEC; (**C**) *Salmonella*; (**D**) *Klebsiella*; (**E**) *Enterococcus*; (**F**) *Proteus*; (**G**) *Staphylococcus*; (**H**) *Streptococcus.* Results are expressed as mean ± SEM from 3 wells. ++ signifies *p* < 0.01 vs. non treated (without bacteria or test product) control and * & ** signify *p* < 0.05 & 0.01 vs. bacteria alone.

**Figure 5:**
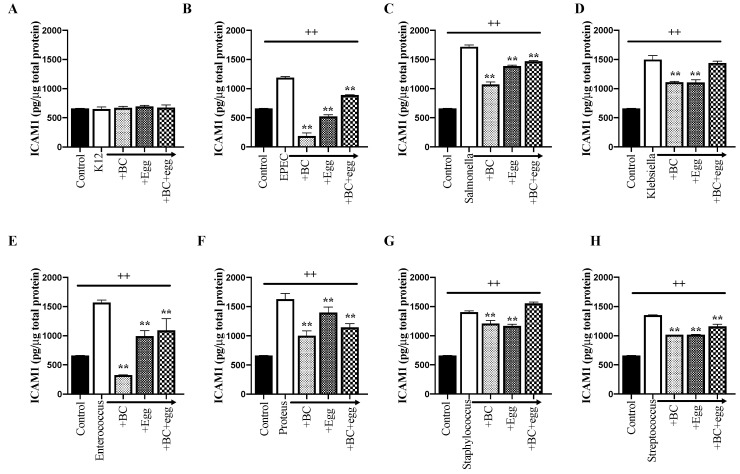
Effect of BC, egg, or the combination on ICAM-1. Bacterial strains (1 × 10^6^ CFU/well) ± BC (1 mg/mL), egg (1 mg/mL), or the combination (0.6 mg/mL BC + 0.4 mg/mL egg) were added to confluent monolayers of Caco-2 cells. Cleared lysates were prepared 24 h later. Control wells received no bacteria or test product. (**A**) *E. coli* K12 (negative control); (**B**) EPEC; (**C**) *Salmonella*; (**D**) *Klebsiella*; (**E**) *Enterococcus*; (**F**) *Proteus*; (**G**) *Staphylococcus*; (**H**) *Streptococcus.* Results are expressed as mean ± SEM from 3 wells. ++ signifies *p* < 0.01 vs. non treated (without bacteria or test product) control and ** signifies *p* < 0.01 vs. bacteria alone.

Figure 2A demonstrates the effects of BC, egg, or the combination on *E. coli* ATCC 25922 O6 induced changes in TEER. The other measured parameters using this bacterium are also shown in Figure 2B–F as an exemplar, so that inter-related mechanisms (such as TEER and tight junction proteins for mucosal integrity, caspase-3, Baxα, and Bcl2 for apoptosis) can be seen easier. As similar findings were found using the other bacteria, their results are presented in Table 1, Table 2 and Table 3 and Figure 3, Figure 4 and Figure 5 and differences to *E. coli* ATCC 25922 O6 noted by exception within the text.

The presence of BC, egg, or the combination without bacteria did not affect TEER. Similarly, noninvasive *Escherichia coli* K12 did not affect TEER. The addition of *E. coli* ATCC 25922 O6 caused a 42% reduction in TEER (Figure 2A) and this reduction was truncated by about 23% adding BC alone, 32% using egg alone and 16% using BC and egg combination (Figure 2A). All other bacterial strains resulted in a similar fall in TEER to using *E. coli* ATCC 25922 O6 (average reduction 133 ± 15.8 Ohms/cm^2^, Table 1). Co-presence of BC alone significantly truncated the fall in TEER when tested against EPEC, *Salmonella*, and *Streptococcus* (Table 1). The addition of egg alone also truncated the reduction in TEER caused by *Enterococcus*, *Klebsiella*, *Staphylococcus*, and *Streptococcus* (Table 1). No additional benefit was seen using the combination.

### 3.3. Study Series 3: Mechanisms of Action of BC, Egg, or the Combination for Maintaining Epithelial Integrity

#### 3.3.1. Tight Junction Proteins

Claudin and ZO1, which are both tight junction proteins, reduced in response to the presence of all bacteria tested (except K12 negative control). The presence of BC truncated the fall in ZO1 in all strains except for *Staphylococcus and Streptococcus* and truncated the fall in claudin in all strains except for *Staphylococcus*. Similar effects were seen with egg alone or the combination (Figure 2H,I and Figure 3 and Table 1).

#### 3.3.2. Cell Apoptosis Assays

All bacteria strains (except negative control K12) increased active caspase-3 (Figure 2B and Figure 4), with a mean increase of 0.133 ± 0.007 change in absorbance A405. The co-presence of BC truncated this increase for all strains except for *Klebsiella* and *Streptococcus*. No additional benefits were seen using either egg alone or the combination.

The proapoptotic molecule Baxα increased in response to all bacteria (except K12). As with caspase-3, the addition of BC alone truncated this increase for all strains, except for *Klebsiella* and *Streptococcus*. No additional benefits were seen using either egg alone or the combination (Figure 2C and Table 2)

Anti-apoptotic Bcl2 levels decreased in response to bacteria except for *Klebsiella* and *Streptococcus,* with BC causing similar reciprocal trends in truncation to the effects on caspase-3 and Baxα (Figure 2D and Table 2).

#### 3.3.3. ICAM-1, VEGF, and Hsp70

Expression of ICAM-1 increased in response to presence of all bacteria tested. BC or egg alone reduced ICAM-1 expression for all bacterial strains (Figure 2G and Figure 5).

The presence of most of the tested bacterial strains (*E. coli*, EPEC, *Salmonella*, *Enterococcus*, *Proteus*, *Staphylococcus*, and *Streptococcus),* resulted in increased VEGF expression. BC, egg, or the combination further increased VEGF levels in most of the bacterial strains (*E. coli,* EPEC, *Salmonella*, *Enterococcus*, *Proteus*, and *Klebsiella*) (Figure 2E and Table 3).

Hsp70 levels increased in response to the presence of bacteria for most of the strains tested with BC or egg, causing further increases except for *Klebsiella* and *Staphylococcus* (Figure 2F and Table 3). Additional benefits were not seen using the combination.

## 4. Discussion

BC and egg both contain multiple antimicrobial and immunomodulatory components essential for host development and defense functions. The major form of immunoglobulin in BC is IgG (>80% of total IgG content), with lower amounts of IgA and IgM [25]. BC contains many cytokines of relevance for immune modulation and cellular responses to stressors such as bacterial infection; these cytokines include TNFα, GMCSF, and interleukin (IL)-1β, -6, and -10. BC also contains over twenty different peptide growth factors including EGF and TGFα, members of the TGFβ family, IGF, PDGF, and milk fat globule epidermal growth factor. Oligosaccharides and glycoproteins in BC may also be relevant in reducing binding of bacteria to epithelial cells through acting as competitive inhibitors by mimicking epithelial cell surface carbohydrates. For a recent detailed review of BC and its constituents see [25].

Similarly, egg contains multiple components of potential biological relevance within the egg white and yolk. Proteins with antimicrobial activity include avian beta-defensin, avidin, beta-microseminoprotein-like, cystatin, gallin immunoglobulin Y, lysozyme, ovalbumin, ovoglobulinG2/TENP, ovoinhibitor, ovomucin, ovotransferrin, phosvitin, pleiotrophin, and vitelline membrane outer layer protein 1. Several of these proteins, such as ovotransferrin, ovomucoid, ovomucin hydrolysates, and phosvitin also act as antioxidants. Multiple factors with immune modulatory activity, such as lysozyme and pleiotrophin, have also been identified. In addition, sulfated glycopeptides generated by proteolysis from ovomucin, chalazae, and yolk membrane can stimulate macrophage function and partial digestion of ovotransferrin, and vitellogenin may generate immune modulatory compounds. For a detailed review of the constituents and biological activity of egg see [14].

Passive immunity is predominantly produced by the IgG content in BC and by IgY in egg. Immunization of cows and chickens results in hyperimmune IgG and IgY which have been shown to benefit conditions such as rotavirus infection [16,26]. However, it is important to note that non-hyperimmune “standard” BC or egg also contains IgG/Y directed against a wide spectrum of bacteria relevant for gut health including *Klebsiella, Salmonella pseudomonas, Staphylococci,* and *E. coli*, due to the cows’ and chickens’ natural exposure [27,28,29,30]. The BC used in the current study was collected during the first 24 h post calving, since there is a rapid decline in IgG and other bioactive components after this time [19].

The bacteria tested in the current series of experiments covered the most common aerobic organisms cultured from patients with SIBO. In addition, we included two more toxic bacteria associated with episodic diarrhea, i.e., EPEC and *Salmonella*, to determine if common protective mechanisms were present across bacteria with differing toxicity and because a BC and egg combination had been reported to reduce diarrhea caused by these organisms [18]. The addition of BC, egg, or the combination did not inhibit growth of the bacteria in vitro, which were results in keeping with previous findings that hyperimmune IgY antibodies raised against 078:K80 *E coli*. only inhibited bacterial growth when added at extremely high concentrations (150 mg/mL) [30]. However, this does not mean the IgG and IgY antibodies (or other antimicrobial factors) are irrelevant in vivo. For example, IgG and IgY may have bound to the bacterial cell walls, but as these studies were performed in vitro, the immune effector cells normally present in vivo would not have progressed the microbial killing process. In addition, Igs may have also mediated some of the beneficial effects of BC and egg in the monolayer experiments by reducing adherence of bacteria to the colonic cells and/or reducing translocation through the monolayers by causing bacterial “clumping” through cross linking. 

To test the effects of BC alone and egg alone we used 1 mg/mL, and we used the same final concentration for the combination (i.e., 1 mg/mL total, comprising 0.6 mg/mL BC with 0.4 mg/mL of egg) to examine if synergy occurred with the combination product. We had previously shown that 1 mg/mL was the optimal concentration for BC or egg for inducing proliferation and migration in a variety of gut cell lines including Caco-2 cells, without causing any toxic effects [17]. Similarly, the 60:40 BC/egg ratio was used as it had been shown to reduce duration of diarrhea in patients with infectious diarrhea [18] and we previously showed synergistic responses when used in this ratio for reducing DSS-induced colitis and NSAID-induced small intestinal injury [17].

Our findings that the noninvasive K12 did not result in changes in TEER, translocation, or any of the other markers established that the findings using the other bacteria were not simply due to a generic “bacterial presence” effect. The co-addition of BC ± egg with *E. coli* K12 also did not affect any of the measured parameters, which was in agreement with our previous findings that BC added to “non-stressed” colonic HT29 cells did not affect caspase-3, Baxα, and Bcl2 levels or affect TEER [24]. When all the other bacteria were assessed, each strain caused a similar fall in TEER. In contrast, there was wide range in the amount of bacterial translocation across the monolayers, which was dependent on the strain tested. Therefore, there are advantages in using more than one method to determine change in mucosal integrity in response to bacterial stress. Beneficial effects of BC alone, or egg alone, were demonstrated using both methods of assessment. Although no synergistic advantage was seen using the BC and egg combination in this in vitro model, this may be because it did not contain the multiplicity of immune cells present in vivo. SIBO is associated with small intestinal inflammatory changes [7] and our previous in vivo studies have shown synergistic anti-inflammatory reparative effects using the combination for reducing both small intestinal and colonic injury [17].

Intestinal epithelial tight junctions are multiprotein complexes that act as selective barriers. There are at least 40 different proteins composing the tight junctions, consisting of both transmembrane and cytoplasmic proteins. We measured claudin-1 as an example of a major transmembrane protein (further notable members being occludin and claudins) and also measured changes in ZO1 which is located on the intracellular side of plasma membrane and anchors the strands to the actin component of the cytoskeleton. The reduction in ZO1 and claudin-1 levels in response to bacterial administration is likely relevant to an explanation for the lowered intestinal integrity and increased permeability of the monolayers. Our finding that BC ± egg truncated these changes may, therefore, have contributed to the enhanced integrity and improvement in TEER.

Increased apoptosis occurred in response to all bacterial strains, with the increase in the pro-apoptotic Baxα, and reduction in anti-apoptotic Bcl-2 signaling molecules, probably contributing to this response. Our finding that BC ± egg reversed changes in Baxα and Bcl2 suggests this pathway is important in mediating their protective effects.

Hsp70 is a protein that protects against excessive against apoptosis [31] and has overlapping protective effects with VEGF which reduces apoptosis, stimulates angiogenesis, and causes immune modulation [32]. These proteins both increased in response to the presence of most of the bacteria tested, with further increases in expression if BC ± egg was co-present, suggesting that (in bacteria where this occurred) stimulation of Hsp70 and VEGF expression may have relevance to the protective effects of BC ± egg.

For all measured parameters, there were variations in the degree of response dependent on the bacterial strain used, or when BC ± egg was added. This is unsurprising given that the processes by which the bacterial strain interacts/injures the epithelium vary depending on the bacteria being assessed. For example, *Klebsiella* did not cause a rise in VEGF when added on its own, or when the combination, BC ± egg, was present. A detailed examination of why these differences occur goes beyond the scope of this manuscript, but it is notable that *Klebsiella* translocate across gut monolayers via transcellular processes, as opposed to the normal paracellular pathways used by other strains [33]. As a further example of variations in mechanisms of action among strains, *S. aureus* releases alpha-toxin which binds to surface receptors on target cells causing formation of transmembrane pores, resulting in increased cytosolic calcium levels, with the toxins also causing downregulation of tight junction proteins including ZO1 [34].

ICAM-1 stabilizes cell–cell interactions and facilitates leukocyte endothelial transmigration. Its upregulation in cells in response to the presence of bacteria would result in increased leukocyte infiltration in vivo but was not be directly demonstrated in the in vitro model used. Our finding that BC ± egg reduced ICAM-1 expression could potentially have beneficial effects if reproduced in the in vivo situation.

Nutraceuticals, also known as functional foods, are products derived from food sources that provide extra health benefits, in addition to their basic nutritional value. Pharmaceuticals usually involve a single chemical molecule which can be directly held responsible for the pharmacological actions they induce. The identification of the relevant compound(s) in nutraceuticals is more difficult as they are less refined and contain multiple potentially important components. However, provided that robust scientific studies are performed, their biological (and clinical) significance remains valid. There is significant consumer interest for such products, due in part to concerns over the risks and side effects of pharmaceutical agents. BC provides a strong example of an evidence-based nutraceutical with over 6000 preclinical and clinical studies having been published. The public perceive BC as the comprehensive “superfood”, linking it to nature’s first food from breastfeeding, with the added advantage of limiting its own digestion when taken orally.

Our previous studies on bioactive components of BC and egg suggest that for both products, the EGFR pathways is important for mediating many of their protective activities. Examples involving gastrointestinal cell lines include the findings that the protective effect of BC against heat-induced apoptosis, and the pro-proliferative and migratory activity of egg were all reduced if an EGFR blocker was administered [17,24]. In addition, EGF induces changes in the composition of tight junctions through activating several signaling pathways such as PKC, MAPK, and STATs. For a detailed review see Tang et al. [35].

TGFβ is also an important molecule for mediating the promigratory activity of BC or egg on gut cell lines [17,19] and is involved in multiple homeostatic pathways including cell motility, immune responses, and mucosal integrity [36]. Further studies would be required to determine the contribution of individual components present in BC and egg that mediate these effects, although the situation is more complex in vivo, as cells are simultaneously exposed to multiple factors that can result in synergistic responses. For example, when bovine lactoferrin and EGF (both present in BC) were added together to rat intestinal IEC-18 cells, it resulted in a synergistic growth response [37].

In conclusion, using an in vitro model, our studies showed BC ± egg strengthened mucosal integrity against a battery of bacteria relevant for SIBO and for infectious diarrhea. Actions included reducing bacterial translocation and apoptosis and enhancing Hsp70 and cell adhesion molecules. These studies support the potential value of BC ± egg for the treatment of these conditions and may have particular value for SIBO where definitive eradication of precipitating organisms may be difficult to achieve. They may also be relevant in explaining the protective effect of BC ± egg against NSAID-induced small intestinal injury [17], where induction of intestinal dysbiosis plays an important role in its pathogenesis [38]. Clinical studies comprising BC ± egg given alone or in combination with probiotics (as oligosaccharides and glycoproteins in BC also possess prebiotic activity [25]), or with other factors to enhance activity, appear warranted.

## Figures and Tables

**Table 1:** Effects of BC alone, egg, or the combination on bacteria-induced changes in TEER and tight junction protein ZO1.

	+Bacteria	Bacteria + BC	Bacteria + Egg	Bacteria + BC + Egg
**TEER (Ohm/cm^2^) Baseline without bacteria control value = 346 ± 5.9**				
*E. coli* K12 (-ve control)	336 ± 10	353 ± 7	374 ± 12	356 ± 11
EPEC	167 ± 6 ++	201 ± 3 ++ **	220 ± 2 ++ **	195 ± 5 ++ **
*Salmonella*	173 ± 2 ++	197 ± 6 ++ **	229.5 ± 4 ++ **	186 ± 1 ++ **
*Klebsiella*	236 ± 10 ++	230 ± 6 ++	259 ± 1 ++ **	216 ± 9 ++
*Enterococcus*	219 ± 3 ++	219 ± 5 ++	231 ± 2 ++ **	220 ± 2 ++
*Proteus*	213 ± 8 ++	227 ± 2 ++	229.8 ± 1 ++	223 ± 1 ++
*Staphylococcus*	281 ± 5 ++	306 ± 14 ++	325 ± 6.8 **	291 ± 3 ++
*Streptococcus*	279 ± 6 ++	326 ± 16 *	323 ± 19 *	290 ± 3 ++
**ZO1 (pg/μg protein) Baseline without bacteria control value = 207 ± 5**				
*E. coli* K12	206 ± 3.5	205 ± 3	206 ± 1	204 ± 1
EPEC	156 ± 6 ++	205 ± 1 ++ **	201 ± 2 ++ **	183 ± 3 ++ **
*Salmonella*	177 ± 8 ++	205 ± 2 ++ **	201 ± 2 ++ **	171 ± 27 ++
*Klebsiella*	151 ± 1 ++	175 ± 0.5 ++ **	163 ± 4 ++ **	153 ± 2 ++
*Enterococcus*	71 ± 21.5 ++	190 ± 2 ++ **	183 ± 0.1 ++ **	175 ± 3 ++ **
*Proteus*	95 ± 1 ++	146 ± 1 ++ **	132 ± 5 ++ **	133 ± 4 ++ **
*Staphylococcus*	161 ± 2 ++	162 ± 1 ++	171 ± 1 ++	155 ± 30 ++
*Streptococcus*	173 ± 1 ++	156 ± 5 ++	175 ± 1 ++	165 ± 28 ++

Bacterial strains ± BC, egg, or the combination were added to apical side of confluent Caco-2 monolayers; 1 mg/mL of BC alone, 1 mg/mL egg alone or 0.6 mg/mL BC + 0.4 mg/mL egg were used. Then, 24 h later, TEER changes were assessed and Caco-2 cell lysates were analyzed for ZO1. Results expressed as mean ± SEM from 3 wells. ++ signifies *p* < 0.01 vs. non treated (without bacteria or test product) control and * & ** signify *p* < 0.05 & 0.01 vs. bacteria alone. *E. coli* K12 is nonpathogenic and noninvasive and used as a negative control.

**Table 2:** Effect of BC alone, egg, or the combination on bacteria-induced changes in apoptosis signaling molecules.

	+Bacteria	Bacteria + BC	Bacteria + Egg	Bacteria + BC + Egg
**Baxα (pg/μg protein) Baseline without bacteria control value = 12.17 ± 1.67**				
*E. coli* K12	14.2 ± 1.67	12.8 ± 2	14.67 ± 1.5	12.3 ± 1.5
EPEC	49.8 ± 0.33 ++	36.7 ± 1.17 ++ **	36.5 ± 0.33 ++ **	44.3 ± 1.16 ++ **
*Salmonella*	44.3 ± 1.17 ++	36.5 ± 1 ++ **	36.5 ± 1 ++ **	44 ± 0.5 ++
*Klebsiella*	63.3 ± 1.17 ++	61.2 ± 1.33 ++	63.5 ± 1.76 ++	63.8 ± 3 ++
*Enterococcus*	67 ± 0.83 ++	37.7 ± 0.83 ++ **	39.3 ± 2.5 ++ **	46.2 ± 2.3 ++ **
*Proteus*	66 ± 0.17 ++	58.8 ± 0.33 ++ **	59.7 ± 0.5 ++ **	64.7 ± 0.8 ++
*Staphylococcus*	55.3 ± 0.83 ++	39.2 ± 1 ++ **	45.8 ± 2.7 ++ **	53.3 ± 3.5 ++
*Streptococcus*	66.8 ± 0.33 ++	64.5 ± 3.3 ++	73.6 ± 2.7 ++	69.3 ± 1.17 ++
**Bcl2 (pg/μg protein) Baseline without bacteria control value = 61.5 ± 0.87**				
*E. coli* K12	59.5 ± 0.3	61.5 ± 0.3	54.2 ± 1.7	56 ± 5.2
EPEC	45 ± 4 ++	61.5 ± 0.9 **	69.5 ± 1.4 ++ **	53 ± 2.9 ++ **
*Salmonella*	38 ± 4.6 ++	59.5 ± 0.9 **	57 ± 3.5 **	54 ± 5.2 **
*Klebsiella*	66 ± 3.5	55 ± 2.6	61.5 ± 2	57 ± 0.6
*Enterococcus*	37.5 ± 0.9 ++	36 ± 0.6 ++	38.5 ± 3.2 ++	35 ± 3.5 ++
*Proteus*	31.5 ± 0.3 ++	61.5 ± 0.3 **	53 ± 4 **	67 ± 3.5 **
*Staphylococcus*	54 ± 7.5	62 ± 5.8	72.5 ± 3.7 ++ *	79.5 ± 6.6 ++ **
*Streptococcus*	63 ± 3.5	51.5 ± 2.6	66.5 ± 0.9 ++ **	81.8 ± 2.2 ++ **

Same experiment as Table 1**,** i.e., 1 mg/mL of BC alone, 1 mg/mL egg alone, or 0.6 mg/mL BC + 0.4 mg/mL egg were used. Results are expressed as mean ± SEM from 3 wells. ++ signifies *p* < 0.01 vs. non treated (without bacteria or test product) control and * or ** signifies *p* < 0.05 or *p* < 0.01 vs. bacteria alone, respectively. *E. coli* K12 is nonpathogenic and noninvasive and used as a negative control.

**Table 3:** Effect of BC alone, egg, or the combination on damaging effect of bacterial strains on VEGF and Hsp70.

	+Bacteria	Bacteria + BC	Bacteria + Egg	Bacteria + BC + Egg
**VEGF (pg/μg protein) Baseline without bacteria control value = 2.45 ± 0.03**				
*E. coli* K12	2.67 ± 0.55	2.57 ± 0.004	2.56 ± 0.01	2.79 ± 0.24
EPEC	2.52 ± 0.05	3.72 ± 0.03 ++ **	3.49 ± 0.01 ++ **	3.41 ± 0.04 ++ **
*Salmonella*	4.4 ± 0.03 ++	5.56 ± 0.01 ++ **	5.2 ± 0.02 ++ **	4.5 ± 0.07 ++ **
*Klebsiella*	2.31 ± 0.36	2.57 ± 0.3	2.62 ± 0.27	3.59 ± 0.26 ++ **
*Enterococcus*	5.11 ± 0.14 ++	5.48 ± 0.15 ++ **	5 ± 0.2 ++	4.8 ± 0.01 ++
*Proteus*	3.16 ± 0.01 ++	3.69 ± 0.14 ++ **	3.21 ± 0.19 ++	3.15 ± 0.01 ++
*Staphylococcus*	4.51 ± 0.06 ++	4.59 ± 0.11 ++	4.53 ± 0.04 ++	4.29 ± 0.29 ++
*Streptococcus*	4.96 ± 0.04 ++	4.58 ± 0.12 ++	4.46 ± 0.01 ++	4.75 ± 0.18 ++
**Hsp70 (pg/μg protein) Baseline without bacteria control value = 566 ± 90**				
*E. coli* K12	675 ± 57	682 ± 30	694 ± 72	677 ± 65
EPEC	492 ± 60	867 ± 87 ++ **	792 ± 40 ++ **	782 ± 4 ++ **
*Salmonella*	642 ± 40	966 ± 12 ++ **	1091 ± 23 ++ **	923 ± 119 ++ **
*Klebsiella*	1396 ± 10 ++	1167 ± 25 ++	1422 ± 28 ++	1234 ± 2 ++
*Enterococcus*	1195 ± 129 ++	1589 ± 15 ++ **	1599 ± 67 ++ **	1425 ± 5 ++ **
*Proteus*	860 ± 28 ++	1083 ± 27 ++ **	1135 ± 11 ++ **	963 ± 21 ++ **
*Staphylococcus*	1172 ± 82 ++	1165 ± 185 ++	1031 ± 101 ++	1185 ± 147 ++
*Streptococcus*	1280 ± 86 ++	1540 ± 10 ++ **	1333 ± 97 ++	1157 ± 235 ++

1 mg/mL of BC alone, 1 mg/mL egg alone, or 0.6 mg/mL BC + 0.4 mg/mL egg were tested. Results are expressed as mean ± SEM from 3 wells. ++ signifies *p* < 0.01 vs. non treated (without bacteria or test product) control and ** signifies *p* < 0.01 vs. bacteria alone.

## Data Availability

Articles are licensed under an open access Creative Commons CC BY 4.0 license, meaning that anyone may download and read the paper for free.

## Supplementary Materials

The following are available online at https://www.mdpi.com/2072-6643/13/3/1024/s1, Table S1. Effect of BC, egg, or the combination on bacterial growth.

## Abbreviations

ANOVA	Analysis of variance
BC	Bovine colostrum
EGF	Epidermal growth factor
EPEC	Enteropathogenic *Escherichia coli*
IGF	Insulin-like growth factor
SIBO	Small intestinal bacterial overgrowth
TEER	Transepithelial electrical resistance
TGF	Transforming growth factor
