# Supplementary material for: Effects of Bovine Colostrum with or without Egg on In Vitro Bacterial-Induced Intestinal Damage with Relevance for SIBO and Infectious Diarrhea
Source: Nutrients. 2021 Mar 22;13(3):1024. doi: 10.3390/nu13031024 (PMC8004259; doi:10.3390/nu13031024)
Supplement: Supplementary file 1 [file nutrients-13-01024-s001.pdf]

| Proliferation (CFU/ml) X$10^5$ | + bacteria | +bacteria + BC | +bacteria +egg | +bacteria +BC+egg |
|---|---|---|---|---|
| *E.coli* K12 | 54.3 +/- 1.3 | 50.7 +/- 2.4 | 54.0 +/- 3.2 | 50.0 +/- 2.5 |
| *E.coli* | 55.3 +/- 0.8 | 50.2 +/- 2.1 | 56.5 +/- 3.3 | 50.3 +/- 2.2 |
| EPEC | 56.5 +/- 2.5 | 57.2 +/- 2.8 | 57.5 +/- 1.3 | 57.7 +/- 2.6 |
| *Salmonella* | 59.5 +/- 2.3 | 61.5 +/- 1.8 | 61.3 +/- 3.2 | 60.3 +/- 1.9 |
| *Klebsiella* | 59.3 +/- 0.8 | 61.7 +/- 2.2 | 61.5 +/- 2.5 | 61.7 +/- 1.1 |
| *Enterococcus* | 62.7 +/- 2.0 | 63.5 +/- 0.6 | 63.7 +/- 0.9 | 62.7 +/- 0.6 |
| *Proteus* | 51.7 +/- 1.9 | 52.3 +/- 1.2 | 52.3 +/- 0.6 | 51.5 +/- 0.8 |
| *Staphylococcus* | 64.2 +/- 0.8 | 61.3 +/- 2.4 | 65.3 +/- 2.2 | 62.0 +/- 2.6 |
| *Streptococcus* | 43.2 +/- 1.5 | 40.8 +/- 1.4 | 44.5 +/- 0.8 | 40.2 +/- 1.2 |

**Supplemental Table 1. (Lack of) effect of Bovine Colostrum (BC), egg or BC+egg combination on bacterial growth.**
Data shown for 24h timepoint using 5 mg/ml of BC alone, 5 mg/ml egg alone or same final concentrate (5 mg/ml) of a 60:40 BC:egg mixture. Results expressed as mean +/- SEM of 6 wells.
